# Quantification of onion (*Allium cepa* L.) evapotranspiration and crop coefficient via weighable lysimeter under semi-arid climate of Melkasa, Ethiopia

**DOI:** 10.1016/j.heliyon.2025.e42566

**Published:** 2025-02-08

**Authors:** Nigusie Kebede, Mekonen Ayana, Boja Mekonnen

**Affiliations:** Department of Water Resources Engineering, Adama Science and Technology University Adama, Ethiopia

**Keywords:** Evapotranspiration, Crop coefficient, Semi-arid, Weighable lysimeter, Onion

## Abstract

In response to the growing challenge of water scarcity in agriculture, efficient irrigation planning and management have become increasingly essential. A critical component of this is determining the precise water requirements of crops. This study was conducted at Melkasa Agricultural Research Centre in Ethiopia during February to May in 2023 and 2024 and focused on quantifying the water requirement and crop coefficient of onion (Allium cepa L.), Nafis variety. Using weighable lysimeters, the study measured field-level water balance components, with crop evapotranspiration calculated through the water balance equation. The CROPWAT model and meteorological data from a station located nearby were used to estimate reference evapotranspiration. The results indicated that the pooled seasonal crop evapotranspiration was 460.27 mm, while the reference evapotranspiration for the same period was 509.18 mm. The average crop coefficient values were determined to be 0.68, 0.89, 1.03, and 0.86 for the initial, developmental, mid-, and late-season stages, respectively. Additionally, a third-order polynomial equation was established to predict the values of the crop coefficient based on the number of days after transplanting. These findings offer valuable insights for improving water resource use efficiency and optimizing irrigation scheduling for the production of onion, particularly in regions like Melkasa, where neither field-based measured data nor site-specific validated estimation methods are lacking.

## 1 Introduction

In many regions, especially in developing countries, the water available for irrigation is decreasing continuously. One key factor is the growing competition for water among various sectors, including domestic and industrial uses [1]. On the other hand, the development of new water resources is constrained by several technical and economic factors, including high costs, a lack of suitable reservoir locations, complexities in water delivery, and environmental concerns [2]. Additional challenges like desertification and the overexploitation of existing water resources [3] further exacerbate the situation. Moreover, climate change impacts on the availability of water resources for agriculture are already visible in many regions, especially arid and semi-arid areas [4]. Furthermore, low efficiencies in irrigated agriculture contribute to increased water demand for irrigation [5], further exacerbating water scarcity.

Thus, under water scarcity conditions for agriculture, proper use and management of available water for irrigation is crucial. In line with this, effective planning and management of irrigation and improving irrigation efficiency are vital. For this purpose, accurate determination of crop water requirements is needed [6,7].

Although measurement of crop evapotranspiration using a weighable lysimeter is costly and time-consuming, it is still useful for calibration of empirical equations. Allen et al. [8] describe a generally accepted procedure for estimating the water requirements of crops for irrigation scheduling. Using lysimeters to measure water balance components is an old approach and yet a helpful method to quantify water requirements of crops directly and drive crop coefficients, which are critical for effective management of irrigation [9]. It uses cropped lysimeters to measure the amount of lost water as a result of transpiration, drainage, and evaporation. These data are then used to calibrate the crop coefficient values [10] and develop area-specific crop coefficients to predict the evapotranspiration of crops from reference evapotranspiration [11].

It is crucial to develop site-specific crop coefficients for accurate determination of crop water requirements under particular climatic conditions. This is because crop water requirements and, hence, crop coefficients vary from location to location depending on factors like local climate, soil types, irrigation regime, management practices, and other environmental factors for the same crop [8,12]. However, such measurements using lysimeters are rarely undertaken due to the requirement of high cost and lack of equipment and laboratory facilities in many areas [13].

When direct measurement is not possible, the indirect method, which uses the Kc approach, in which crop water requirement is estimated as a product of crop coefficient and reference evapotranspiration, is used. In this approach, the accuracy of the water requirement determined depends on the method used to estimate the reference evapotranspiration.

Onion is among the most widely cultivated vegetable crops in Ethiopia, particularly in the study area. The study area has historically been known for its limited water resources and inefficient irrigation water management. Currently, the area is facing significant water stress due to demand for water exceeding supply [14], underscoring the crucial need for precise irrigation management practices. Despite the importance of onion crops, their crop coefficient and water requirements remain insufficiently studied.

Several studies have been conducted to estimate ETc and Kc for onion in various regions with semi-arid climate conditions worldwide [4,[15], [16], [17]]. However, some studies have been conducted to determine the water use of onion in Ethiopia. Bossie et al. [18], Dirirsa et al. [19], and Abebe et al. [20] were among those who offered insights into crop coefficients and crop water requirements for the Red Bombay onion variety. Another improved onion variety that is widely grown in the area is the Nafis onion variety. This variety of onion is preferred by most farmers due to its early maturing nature, shorter production time, and hence lower production costs. However, the crop coefficient and water requirement are not yet studied.

In light of this, this region has historically been known for its limited water resources and inefficient irrigation water management. The aim of this study was to determine the crop coefficient and water requirement of onion using a weighable lysimeter in order to optimize irrigation water use and enhance water use efficiency for onion production in semi-arid regions of Melkasa, Ethiopia.

## 2 Materials and methods

### 2.1 Study area description

**Fig. 1:**
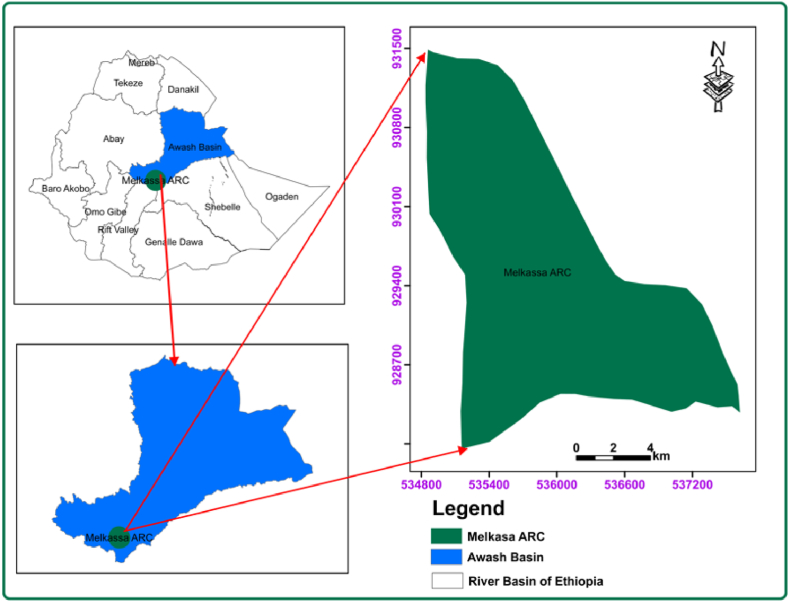
Map of the study area.

The experimental study was conducted for two consecutive years (2023 and 2024) at Melkasa Agricultural Research Centre (MARC). The center is located at 8° 24′ latitude and 39° 21′ longitude, with an altitude of 1550 m above sea level. Agro-ecologically, the area is classified as a semi-arid zone, in which the climatic condition is characterized as hot throughout the year with an annual average rainfall of 845.5 mm, 14 °C minimum and 29 °C maximum temperatures. The study area location map is illustrated in Fig. 1.

### 2.2 Experimental setup

**Fig. 2:**
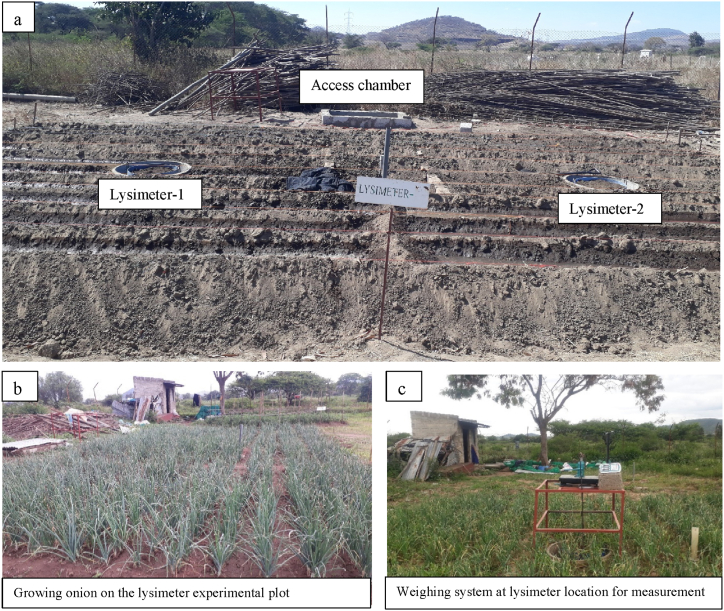
The experimental lysimeter's layout (a) and field condition pictures (b and c).

For determining the crop coefficient, Kc and water requirement, ETc of onion, a weighable type of lysimeter designed, constructed, and tested by Kebede et al. [9] was used. The lysimeters are circular-shaped, 65 cm in depth (including the depth of the rim and drainage material, 15 cm), and 55 cm in diameter (yielding 0.24 m^2^ of internal surface area) each, but for buffering conditions, onion was planted on a 10 m by 6 m area outside of the lysimeters. The two lysimeters are located 4 m apart from each other within the buffer zone. An access chamber with 1.5, 1.2, and 1.4 m of length, width, and depth, respectively, supported with a ladder located at a side of the lysimeters, was constructed, enabling the measurement of drained water from each lysimeter, which was directed via drainage pipes towards the drained water collector. The arrangement of the lysimeter experimental plot is shown in Fig. 2a, while Fig. 2b and c depict the field conditions, specifically the growing onion and the weighing system at the lysimeter location for measurement, respectively. A detailed description of the weighable lysimeter is available in Kebede et al. [9].

The onion variety considered in this study was Nafis, an improved variety widely cultivated in the region. Moreover, this variety is highly preferred by most farmers due to its early maturity, which allows for faster harvesting, a shorter production cycle, and reduced production costs. However, despite its popularity and the economic advantages it offers, essential parameters useful for irrigation management, such as its crop coefficient and water requirements, are insufficiently studied, highlighting the need for further research.

The characteristics of the experimental field soil were analysed in Melkasa Agricultural Research Center soil laboratory. The parameters analysed include bulk density, soil texture, moisture content at wilting point, and field capacity by taking soil samples at intervals of 15 cm. The result revealed that the texture of clay loam with 1.2 g cm^−3^ bulk density and volume-based soil moisture content of 26.25 % and 38.88 % at the permanent wilting point (PWP) and field capacity (FC), respectively.

### 2.3 Crop agronomy

Seedlings of onion, Nafis variety, were prepared according to the way the farmers actually practice to supply as planting material. The experimental plot was prepared with 60 cm-spaced ridges by aligning one ridge to be at the center of the lysimeters. On February 1, in both years, 2023 and 2024, of the growing seasons, the seedlings were transplanted in a single row on each ridgeside spaced 20 cm apart, providing 10 cm of space between plants. All recommended agronomic practices adopted were the same for both the buffer area as well as in the lysimeters. Fertilizers with 100 kg and 200 kg per hectare rates of Urea and DAP, respectively, were used as nutrient sources in order to improve the development and productivity of onion crops. It was harvested on May 21 during the growing season of 2023 and May 20 during the growing season of 2024. The total growth season was 110 days, which is the sum of 15, 30, 40, and 25 days, respectively, for the growth stages: initial, developmental, mid-, and late-season stages.

### 2.4 Soil moisture monitoring and application of irrigation

The change in soil moisture content was measured directly from the mass change of the lysimeter between two time intervals [21,22]. At the beginning, the moisture of the soil was placed at its field capacity, then the change in moisture content between irrigation intervals was monitored as a difference in mass of the lysimeter at the current and previous time before irrigation and computed using Equation (1).

(1) $$\Delta SM={WL}_{t}-{WL}_{t-1}$$

$$Where,\Delta SM=Change\;in\;soil\;Moisture\;content\;\left(kg\right),{WL}_{t}=Weight\;of\;lysimeter\;at\;current\;time\;\left(kg\right),{WL}_{t-1}=Weight\;of\;lysimeter\;at\;previous\;time\;\left(kg\right)\text{,}$$

Irrigation water was applied at or before the depletion factor (p) reached 0.3, as suggested by Allen et al. [8], to establish an optimal soil moisture condition. This was achieved through continuous soil moisture analysis, which was undertaken at 2- to 3-days intervals to ensure the availability of optimum soil moisture content in the root zone. The quantity of water required to revert the soil to field capacity was computed using Equation (2) and applied using a calibrated watering can.

(2) $$SMD={WL}_{FC}-{WL}_{i}$$

$$Where,SMD=Soil\;Moisture\;Depleted\;\left(kg\right),{WL}_{FC}=Weight\;of\;lysimeter\;at\;field\;capacity\;\left(kg\right),{WL}_{i}=Weight\;of\;lysimeter\;at\;urrent\;condition\;\left(kg\right)\text{,}$$

### 2.5 Evapotranspiration determination

The determination of evapotranspiration using the weighable lysimeters involved the measurement of the soil water balance components. Weather data, including rainfall (P), was obtained from Melkassa Agricultural Research Center weather station, which is situated around 50 m away from the lysimeter plot. Applied irrigation water (I) and change in soil moisture content (ΔSM) were quantified, respectively, using Equations (2), (1)). While the drainage water (Dp) was collected at the access chamber and measured.

The evapotranspiration of onion crops for each growth stage or time interval, expressed as unit mass or volume per unit area, or by equivalent water height [23], was computed using Equation (3), the general water balance equation.

(3) $${ET}_{c}=I+P-D_{p}-\Delta SM$$

$$Where,{ET}_{c}=crop\;evapotranspiration\;\left(mm\right),I=applied\;irrigation\;water\left(mm\right),P=rain\;fall\;\left(mm\right),D_{p}=deep\;percolation\left(drained\;water\right)\left(mm\right),\Delta SM=change\;in\;soil\;moisture\;content\left(mm\right)$$

### 2.6 Reference evapotranspiration determination new

Reference evapotranspiration was computed using the version 8.0 CROPWAT model that employs the Penman-Monteith equation [8]. Input climatic data for the model includes temperature (maximum and minimum), relative humidity, wind speed, and sunshine hours, which were collected from the weather station of the research center. The weather station is situated around 50 m from the lysimeter plot and measures various parameters, including temperature with a mercury-in-glass thermometer, relative humidity with psychrometers, wind speed with cup anemometers, sunshine hours with a Campbell-Stokes sunshine recorder, and precipitation with a manual rain gauge.

### 2.7 Crop coefficient determination

The ratio of measured evapotranspiration of crop, ETc to ETo, reference evapotranspiration from version 8.0 of the CROPWAT model, was used to calculate the crop coefficient, or Kc (Equation (4)).

(4) $$K_{c}=\frac{{ET}_{c}}{{ET}_{o}}$$

$$Where,K_{c}=Crop\;coefficient,{ET}_{c}=Crop\;evapotranspiration\;\left(mm\right),and\;{ET}_{o}=Reference\;evapotranspiration\;\left(mm\right)\text{.}$$

## 3 Results and discussions

### 3.1 Reference evapotranspiration, ETo

**Fig. 3:**
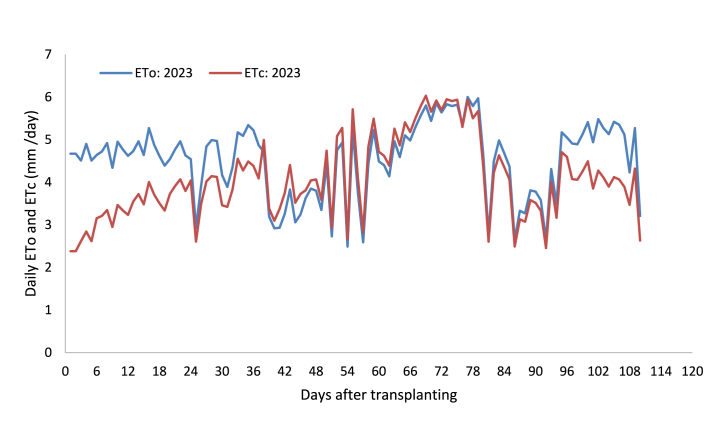
Reference evapotranspiration and onion evapotranspiration during the 2023 growing season.

**Fig. 4:**
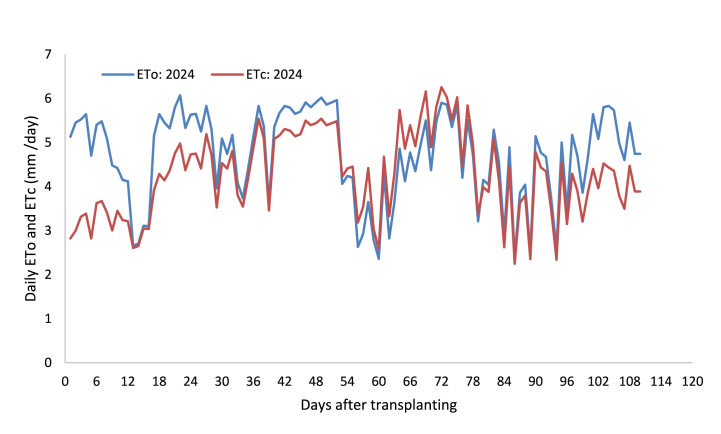
Reference evapotranspiration and onion evapotranspiration during the 2024 growing season.

The daily computed ETo throughout the growing period is presented in Fig. 3, Fig. 4. Table 1 presents the average decadal weather input data for the CROPWAT model and computed reference evapotranspiration (ETo) using the model. During the 2023 growing season, the reference evapotranspiration value fluctuated between 2.49 and 6.00 mm day^−1^ with a 4.50 mm day^−1^ average value, while during the 2024 growing season it fluctuated from 2.36 to 6.07 mm day^−1^ with a 4.22 mm day^−1^ average value. The pooled reference evapotranspiration value for the growth stages: initial, developmental, mid- and late-season was found to be 4.62, 4.74, 4.65, and 4.48 mm day^−1^, respectively (Table 2). The average reference evapotranspiration value for the whole growth season was found to be 509.18 mm.

**Table 1:** Average decadal weather data and computed reference evapotranspiration.

DAT	2023 growing period						2024 growing period					
	Tmin	Tmax	RH	U_2_	Sunshine	ETo	Tmin	Tmax	RH	U_2_	Sunshine	ETo
	°C	°C	%	m s^−1^	hr	mm day^−1^	°C	°C	%	m s^−1^	hr	mm day^−1^
1–10	8.3	29.9	84.5	2.7	10.1	4.68	13.8	29.9	65.4	2.8	9.1	5.13
11–20	9.4	29.3	83.4	3.0	10.1	4.74	14.5	29.9	67.1	1.7	6.1	4.14
21–30	11.2	30.7	83.3	2.1	8.1	4.47	11.5	32.1	58.5	2.0	9.6	5.39
31–40	13.9	30.3	81.2	2.6	7.3	4.48	14.5	30.9	67.3	1.6	8.0	4.74
41–50	14.4	27.3	73.5	1.7	3.9	3.54	13.5	32.3	58.2	2.1	10.1	5.81
51–60	14.2	28.0	71.0	1.8	5.7	4.06	14.6	28.9	71.9	1.6	4.7	3.87
61–70	14.3	29.8	66.6	1.8	8.8	5.03	14.9	29.1	71.2	1.6	6.8	4.37
71–80	14.1	30.9	61.3	2.0	10.5	5.67	14.4	31.2	68.1	1.8	8.3	5.02
81–90	14.6	27.0	76.2	1.2	6.0	3.81	15.6	28.9	73.0	1.3	5.7	3.96
91–100	14.3	28.7	71.7	1.5	7.8	4.46	15.4	29.0	72.7	1.3	7.2	4.26
101–110	14.7	30.6	71.8	2.0	8.7	4.94	15.2	31.7	63.9	1.8	9.3	5.26

DAT - days after transplanting, Tmax - maximum temperature, Tmin - minimum temperature, U₂ - wind speed at 2 m height, RH - relative humidity, and ETo - reference evapotranspiration.

**Table 2:** Stage-based and seasonal reference evapotranspiration.

Growing season	Initial (mm day^−1^)	Developmental (mm day^−1^)	Mid-season (mm day^−1^)	Late season (mm day^−1^)	Seasonal (mm)
2023	4.70	4.32	4.70	4.43	498.78
2024	4.54	5.16	4.59	4.52	519.58
Average	4.62	4.74	4.65	4.48	509.18

### 3.2 Crop evapotranspiration (ETc) of onion

The daily fluctuation in water requirement of onion throughout the growing season is presented in Fig. 3, Fig. 4. Table 3, Table 4 present the average decadal and stage-based evapotranspiration of onion, as quantified from the water balance of the lysimeter. It shows that the evapotranspiration of onion was lower during the initial stage, raised at the development stage, peaked at the mid-season stage, and slowly declined at the late-season stage. The average water requirements of onion for the growth stages: initial, developmental, mid-, and late-season are presented in Table 4. The averaged values varied from 3.12 mm day^−1^ to 4.81 mm day^−1^ at the initial and mid-season growth stages of onion, respectively. The seasonal water requirement for onion was found to be 460.27 mm on average. The low crop evapotranspiration of onion at the late season stage following the initial stage was mainly due to limited canopy cover in the early stage and termination of leaf growth in the late stage [8,19,20]. However, high water requirements were observed at the mid-season stage due to fully developed canopies and processes like flowering, tuber formation, and filling [20].

**Table 3:** Decadal measurements of water balance components and evapotranspiration of onion.

DAT	2023 growing season					2024 growing season				
	I	P	Dp	ΔS	Average ETc	I	P	Dp	ΔS	Average ETc
	mm	mm	mm	mm	mm	mm	mm	mm	mm	mm
1–10	25.52	0.00	0.00	−3.33	2.89	29.06	0.00	0.00	−3.31	3.24
11–20	35.21	0.00	0.00	−0.55	3.58	22.40	20.60	10.08	−1.33	3.42
21–30	25.63	7.29	0.00	−4.56	3.75	32.50	17.00	4.50	−0.78	4.58
31–40	37.40	55.50	32.80	19.69	4.04	20.31	123.50	76.25	22.71	4.49
41–50	0.00	67.92	47.50	−17.72	3.81	28.85	0.00	9.23	−33.70	5.33
51–60	23.54	35.00	17.50	−3.21	4.43	21.88	41.29	10.21	9.52	4.34
61–70	38.13	17.50	4.33	−1.78	5.31	32.92	29.71	6.69	7.41	4.85
71–80	52.40	63.50	33.73	26.59	5.56	34.06	15.00	1.27	−4.81	5.26
81–90	15.00	71.58	64.90	−14.40	3.61	20.83	25.46	7.63	2.08	3.66
91–100	2.19	47.79	25.48	−14.79	3.93	0.00	109.54	83.79	−14.77	4.05
101–110	0.00	5.42	0.00	−33.33	3.88	0.00	0.00	0.00	−40.63	4.06

DAT - days after transplanting, I - irrigation, P - rainfall, Dp - deep percolation, ΔS - change in soil moisture content, and ETc - crop evapotranspiration.

**Table 4:** Growth stage-based and seasonal evapotranspiration of onion (Nafis variety).

Growing season	Initial (mm day^−1^)	Developmental (mm day^−1^)	Mid-season (mm day^−1^)	Late season (mm day^−1^)	Seasonal (mm)
2023	3.08	3.83	4.82	3.75	447.66
2024	3.15	4.54	4.79	3.92	472.88
Average	3.12	4.19	4.81	3.84	460.27

### 3.3 Crop coefficient of onion

**Fig. 5:**
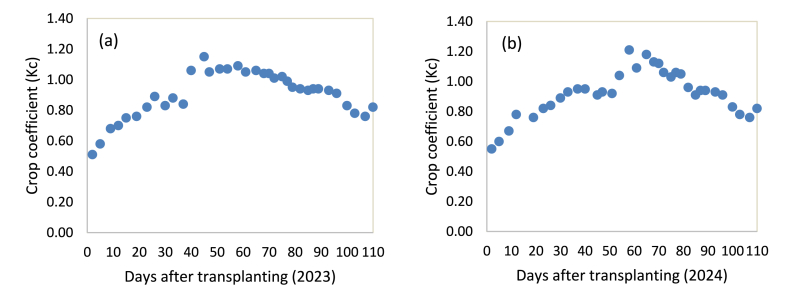
Observed values of onion crop coefficients for the growing seasons of 2023 (a) and 2024 (b).

Decadal and observed values of crop coefficients were established and presented in Table 5 and Fig. 5a and b, respectively. It can be seen that the crop coefficient consistently raised from 0.62 to 1.11 during 10–60 days after transplanting. During 60–80 days, the value was decreased slightly from 1.11 to 1.08 and then to 1.01. During the late season, 80–110 days after transplanting, it further declined to 0.78. Table 6 presents the average growth stage-based crop coefficient. The value increased from 0.68 at the initial to 1.03 at mid-season and then declined to 0.86 at the late-season growing stage. The crop coefficient value reached its maximum at the critical time, approximately covering from 40 to 85 days after transplanting. During the growth stages of development and mid-season, the increased crop coefficient value is a result of rapid crop development [11]. The crop's development and covering of the ground to attain full size with growing root depth and plant height resulted in a rise in water abstraction and an increase in crop evapotranspiration [8,24]. The gradual decline in crop coefficient value during the late-stage season was due to decreased water requirement as a result of termination of leaf growth [8,20].

**Table 5:** Decadal values of crop coefficient.

DAT	2023 growing season			2024 growing season			Average Kc
	ETc	ETo	Kc	ETc	ETo	Kc	
	mm	mm		mm	mm		
1–10	2.89	4.68	0.62	3.24	5.13	0.63	0.62
11–20	3.58	4.74	0.75	3.42	4.14	0.83	0.79
21–30	3.75	4.47	0.84	4.58	5.39	0.85	0.84
31–40	4.04	4.48	0.90	4.49	4.74	0.95	0.92
41–50	3.81	3.54	1.08	5.33	5.81	0.92	1.00
51–60	4.43	4.06	1.09	4.34	3.87	1.13	1.11
61–70	5.31	5.03	1.06	4.85	4.37	1.11	1.08
71–80	5.56	5.67	0.98	5.26	5.02	1.05	1.01
81–90	3.61	3.81	0.95	3.66	3.96	0.92	0.94
91–100	3.93	4.46	0.88	4.05	4.26	0.95	0.92
101–110	3.88	4.94	0.78	4.06	5.26	0.77	0.78

**Table 6:** Growth stage-based values of crop coefficient.

Growing season	Growth stage			
	Initial	Developmental	Mid-season	Late season
2023	0.66	0.89	1.03	0.85
2024	0.69	0.89	1.04	0.87
Average	0.68	0.89	1.03	0.86

The average growth stage-based values of crop coefficient found in the current study were 0.68, 0.89, 1.03, and 0.86, respectively, for the growth stages: initial, developmental, mid-, and late-season. Table 7 illustrates crop coefficient values found in the literature and this study. The values obtained in this study were aligned well with the values reported by Abebe et al. [20], Dirirsa et al. [19], and López-Urrea et al. [15], even if the result of Abebe et al. [20] has a slightly lower value at the initial stage. The report of Hend et al. [16] agrees with this study except for the initial and developmental stages. The crop coefficient value reported by Martín De Santa Olalla et al. [25] comports with the values of the current study, except for the initial and late season stages. The finding of Bossie et al. [18] shows lower values for all growth stages.

**Table 7:** Stage-based onion crop coefficient values determined by different authors compared to the current study.

Author	Growth stage			
	Initial	Developmental	Mid-season	Late season
Abebe et al. [20]	0.49	0.90	1.01	0.79
Hend et al. [16]	0.40	0.41	1.10	0.87
Dirirsa et al. [19]	0.61	0.86	1.02	0.80
Bossie et al. [18]	0.47	–	0.99	0.46
López-Urrea et al. [15]	0.65	–	1.20	0.75
Martín De Santa Olalla et al. [25]	0.50	–	1.00	0.70
Current study	0.68	0.89	1.03	0.86

**Fig. 6:**
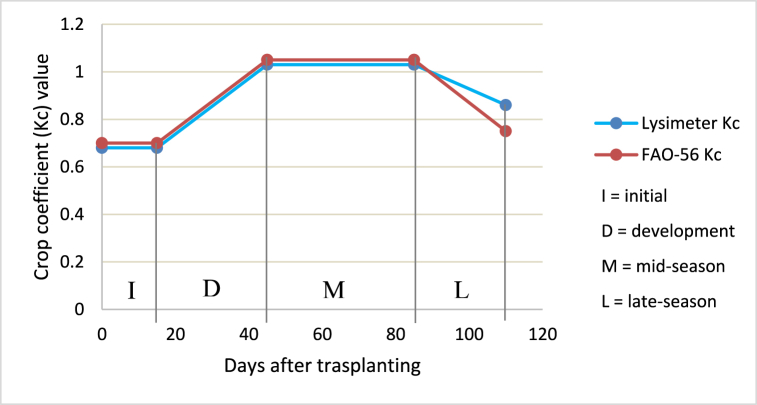
Crop coefficient determined using lysimeter and FAO-56 recommended for onion.

Furthermore, a comparison was made between the crop coefficient values developed using the lysimeter and FAO-56 suggested Kc for onion. The crop coefficient curve derived in this study (Fig. 6) has the same trend as the FAO-56 estimated curve. In comparison, the values were almost similar during the initial, developmental, and mid-season growth stages. However, the crop coefficient value developed using the lysimeter was slightly higher in the late season.

According to Allen et al. [8], it is believed that local differences in environmental factors lead to variance in crop developmental stage and variety selection, which impacts crop coefficient. Key environmental factors include temperature, which affects the rate of plant growth and development; wind speed, which affects evapotranspiration rates; humidity, which influences transpiration and water loss; and solar radiation, which drives photosynthesis and energy absorption. Soil characteristics, such as texture, structure, and moisture-holding capacity, further contribute to these variations by determining how water and nutrients are absorbed by the crops. These factors vary significantly across different geographic regions, creating distinct growing conditions that directly influence the water requirements of crops and their overall performance. These variations clearly demonstrate the challenges associated with employing crop coefficients to estimate agricultural water requirements in a given year with varying crop development patterns, as well as the difficulties in extrapolating crop coefficients to different locations [12].

**Fig. 7:**
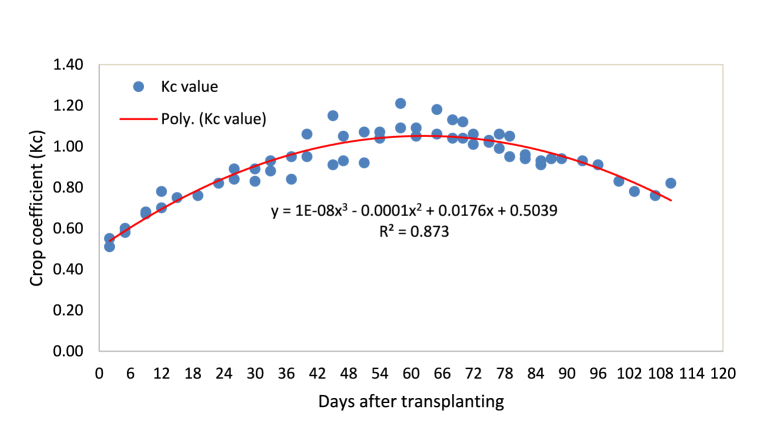
Crop coefficient of onion as a function of number of days after transplanting.

A third-order polynomial equation was fitted to measured data with R^2^ equal to 0.87, as shown in Fig. 7. Different studies proposed a similar third-order polynomial function to predict the variation in the crop coefficient of onion [18,26].

## 4 Conclusion

The water requirements and crop coefficient of the Nafis onion variety were determined through field measurements conducted over two consecutive years in the semi-arid climate of Melkasa, Ethiopia. The daily average evapotranspiration of the onion crop ranged from 3.12 mm at the initial to 4.81 mm at mid-season growth stages. The total seasonal water requirement of the crop was calculated to be 460.27 mm. Crop coefficient values for initial, developmental, mid-, and late-season stages were determined as 0.68, 0.89, 1.03, and 0.86, respectively, aligning well with the standard literature values, including the FAO guidelines. Furthermore, a third-order polynomial equation was presented to predict the values of the crop coefficient. These findings offer a reliable basis for improving irrigation water use efficiency and optimizing irrigation scheduling for the production of onion, particularly in semi-arid regions like Melkasa, where neither field-based measured data nor site-specific validated estimation methods are lacking.

## 5 Recommendation and future work

The lysimeters used in this study were specifically developed for shallow-rooted crops, limiting their applicability for measuring ETc and Kc for deep-rooted crops [9]. Scaling up the size and capacity of lysimeters to accommodate a wider range of crops is a critical consideration, particularly in regions like Ethiopia, where weighing types of lysimeters are not available. Additionally, integrating lysimeter data with remote sensing techniques could significantly enhance the spatial and temporal assessment of ETc and Kc values. This approach could also support the development of ETo maps, providing valuable tools for improved water resource management.

## CRediT authorship contribution statement

**Nigusie Kebede:** Writing – original draft, Methodology, Investigation, Formal analysis, Conceptualization. **Mekonen Ayana:** Writing – review & editing, Supervision, Resources, Methodology, Conceptualization. **Boja Mekonnen:** Writing – review & editing, Supervision, Methodology.

## Data availability statement

Data will be made available on request.

## Declaration of competing interest

The authors declare that they have no known competing financial interests or personal relationships that could have appeared to influence the work reported in this paper.
